# Epidermoid Cyst Arising in the Submandibular Region

**DOI:** 10.1155/2013/419289

**Published:** 2013-09-28

**Authors:** Masanori Kudoh, Hiroyuki Harada, Ken Omura, Yoshimasa Ishii

**Affiliations:** ^1^Division of Oral and Maxillofacial Surgery, Ebina General Hospital, 1320 Kawaraguchi, Ebina, Kanagawa 243-0433, Japan; ^2^Oral and Maxillofacial Surgery, Department of Oral Restitution, Division of Oral Health Sciences, Graduate School, Tokyo Medical and Dental University, Ebina 243-0433, Japan

## Abstract

Dermoid and epidermoid cysts in the oral cavity frequently develop in the midline or sublingual region of the floor of the mouth. Here, we report a rare case of an epidermoid cyst in the submandibular region. The patient was a 69-year-old man with a chief complaint of a mass in the right submandibular region. A mobile, elastic, relatively soft mass without tenderness was palpable in this region. The skin covering the mass was normal. MRI showed a cystic lesion measuring 3.5 × 3.0 cm under the platysma in the right submandibular region. Cystectomy was performed under general anesthesia. There was no adhesion to surrounding tissue and the right submandibular gland was preserved. The surgical specimen was cystic and contained soybean cord-like materials. Histopathologically, the cyst wall was lined by stratified squamous epithelium with no skin appendage, suggesting an epidermoid cyst. The postoperative course was uneventful and without recurrence after 28 months.

## 1. Introduction

Dermoid and epidermoid cysts in the oral cavity frequently develop in the midline or sublingual region of the mouth floor [1–6]. In the cervical region, dermoid cysts in the lateral neck have occasionally been reported, but an epidermoid cyst in the submandibular region is very rare. Epidermoid cysts consist of an epithelial-lined wall that may be partly keratinized, with no evidence of skin appendages. In contrast, dermoid cysts are cysts with an epithelial lining enclosing skin appendages such as hair, hair follicles, sebaceous glands, and sweat glands.

Here, we present a rare case of an epidermoid cyst in the submandibular region.

## 2. Report of Case

**Figure 1:**
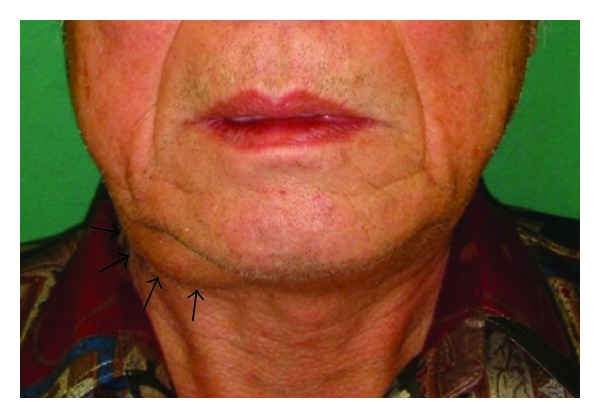
Facial view at the first visit. A mass measuring 4.0 × 3.5 cm was evident in the right submandibular region. This photograph is published with the patient's consent.

A 69-year-old man presented with a 2-year history of a mass in the right submandibular region. He had no symptoms, but had recently noticed the slow enlargement of the mass. He was referred to our hospital in January 2011. He had no significant medical history. Initial examination showed a mobile, elastic, relatively soft mass measuring 4.0 × 3.5 cm in the submandibular region. The mass was not tender and the skin moved freely over the mass (Figure 1). There was no swelling of the floor of the mouth and there was good salivary flow in the right submandibular gland duct.

**Figure 2:**
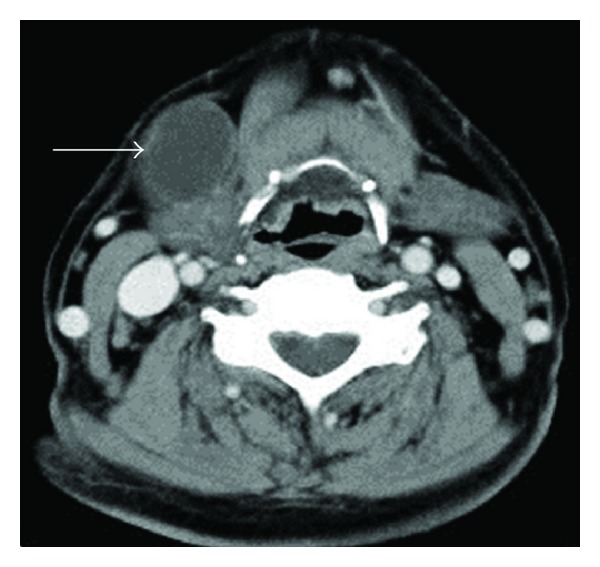
Contrast-enhanced CT (axial view). The cystic lesion was located anterior to the submandibular gland under the platysma and the margin of the mass was enhanced, suggesting a cystic lesion (arrow).

Contrast-enhanced CT showed a mass measuring 3.5 × 3.0 cm with a clear boundary and regular margin positioned under the platysma anterior to the right submandibular gland (Figure 2). The margin was enhanced in a ring pattern. The mass displaced the submandibular gland posteriorly. T2-weighted MRI showed a well-circumscribed cystic lesion with a low-density boundary and heterogeneous inner region. These findings led to diagnosis of a branchial cleft cyst.

Surgical excision was performed under general anesthesia in March 2011. A skin incision parallel to the lower border of the mandible was made above the mass, and then the division was advanced to the lower layer of the platysma and the mass was excised with ease. There was no adhesion with the submandibular gland and it was possible to preserve the gland, including the capsule. The postoperative course was uneventful with no recurrence after 28 months.

**Figure 3:**
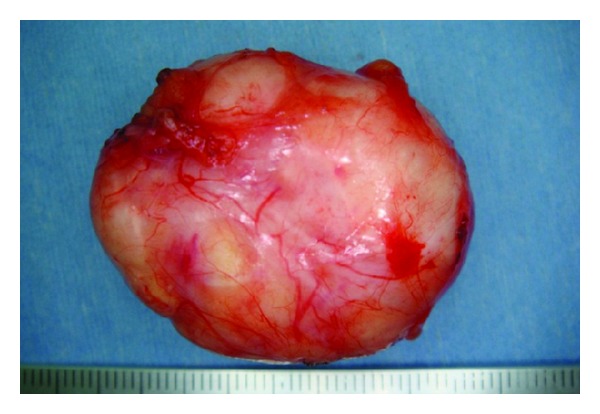
Surgical specimen. The mass was cystic, measured 4.0 × 3.0 × 2.5 cm, and had a smooth surface.

**Figure 4:**
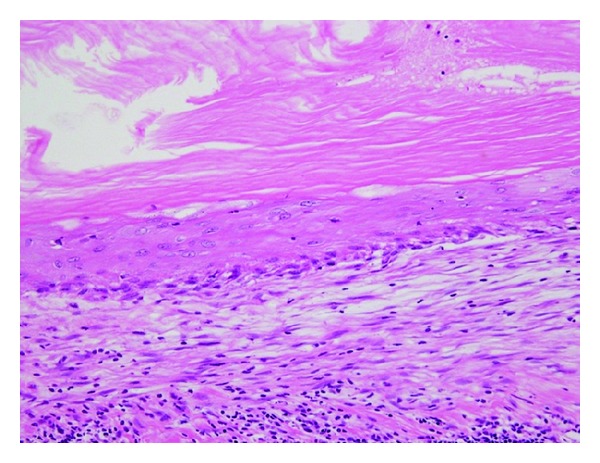
Histopathology of the surgical specimen (H-E ×200). The wall was lined by relatively thin keratinized stratified squamous epithelium and the lumen was filled with soybean cord-like keratinized material.

Macroscopically, the surgical specimen was a cyst of 4.0 × 3.0 × 2.5 cm with a smooth surface (Figure 3) and a relatively thick wall that contained soybean cord-like material. Histopathologically, the cyst wall was lined with a relatively thin keratinized stratified squamous epithelium similar to skin epidermis, and keratohyalin granules were present. The basal layer was flat without rete peg formation. The contents comprised a soybean cord-like keratinized material and the cyst wall included fibrous tissues. The wall had lymphocyte infiltration, but no skin appendage (Figure 4). These features confirmed the diagnosis of an epidermoid cyst.

## 3. Discussion

Dermoid and epidermoid cysts can develop in any region of the body. For congenital cases, the mechanism of development involves embryonal aberration of residual ectodermal tissues, while for acquired cases, the cyst is thought to be derived from epithelial tissue aberration in the dermis due to trauma, inflammation, or surgery [1]. The midline or sublingual region of the floor of the mouth is a common location of cysts in the oral cavity. Lesions in the midline region tend to be congenital and caused by ectodermal tissue in the fused region of the first and second branchial arches. Dermoid and epidermoid cysts have also been described in the submandibular region [2–5], but have all been located between the mylohyoid and hyoglossus muscles, suggesting that the cysts initially arose in the midline region of the floor of the mouth and then moved laterally upon expansion [1–6]. Adhesion and cord-like structures found around the cysts are also suggestive of cyst movement [1–3, 7].

A dermoid or epidermoid cyst arising in the lateral side of the submandibular gland, as in our case, is extremely rare. There has been one previous report of a dermoid cyst [7], which was assumed to have developed in the submandibular space due to ectodermal tissue aberration into the fused region of the first branchial pouch or on the dorsal side of the first branchial arch. In our case, there was no adhesion to surrounding tissues or cord-like structures and the patient had no history of trauma, inflammation, or surgery in the submandibular region. Thus, the case was diagnosed as a congenital epidermoid cyst with the mechanism of development described above. 

Submandibular gland tumor, ranula, malignant lymphoma, metastatic lymph node, hemangioma, and branchial cleft cyst require differentiation from dermoid and epidermoid cysts in the submandibular region. In our case, CT and MRI revealed a cystic lesion located anterior to the submandibular gland. A branchial cleft cyst was suspected based on the frequency of this cyst in the neck although development is usually along the anterior border of the sternocleidomastoid muscle. Diagnosis is difficult using imaging and clinical findings alone, and total excision is required for definite diagnosis. The recurrence rate of an epidermoid cyst is low, but malignant transformation may occur [8]. In our case, there has been no recurrence in 28 months postoperatively.
